# Sequencing and *de novo* Analysis of *Crassostrea angulata* (Fujian Oyster) from 8 Different Developing Phases Using 454 GSFlx

**DOI:** 10.1371/journal.pone.0043653

**Published:** 2012-08-27

**Authors:** Ji Qin, Zixia Huang, Jun Chen, Quan Zou, Weiwei You, Caihuan Ke

**Affiliations:** 1 State Key Laboratory of Marine Environmental Science, Xiamen University, Xiamen, China; 2 College of Ocean and Earth Sciences, Xiamen University, Xiamen, China; 3 College of Information Science and Technology, Xiamen University, Xiamen, China; King Abdullah University of Science and Technology, Saudi Arabia

## Abstract

Research on the mechanism for early development of shellfish, such as body plan, shell formation, settlement and metamorphosis is currently an active research field. However, studies were still limited and not deep enough because of the lack of genomic resources such as genome or transcriptome sequences. In the present research, *de novo* transcriptome sequencing was performed for *Crassostrea angulata*, the most economically important cultured oyster species in China, at eight early developmental stages using the 454 sequencing technology. A total of 555,215 reads were produced with an average length of 309 nucleotides that were then assembled into 10,462 contigs. As determined by GO annotation and KEGG pathway mapping, functional annotation of the unigenes recovered diverse biological functions and processes. Six unique sequences related to settlement, metamorphosis and growth were subsequently analyzed by real-time PCR. Given the lack of whole genome information for oysters, transcriptome and *de novo* analysis of *C. angulata* from the eight different developing phases will provide important and useful information on early development mechanism and help genetic breeding of shellfish.

## Introduction

Lophotrochozoans usually show similar early development processes, but body plan and behavior pattern for the adults are very different. Mollusks are the most diverse animal phylum and the representative lophotrochozoan phylum which exhibits two ancient developmental features: spiral cleavage and trochophore larva. Bivalves, which include clams, oysters, mussels, and scallops, are the second largest group of mollusks [1]. Therefore, research on early developmental mechanism on bivalves will provide useful information on evolution, phylogeny and diversification for lophotrochozoan.

Apart from common developmental issues to the model animals, such as cleavage, gastrulation formation and organogenesis, there are other specific developmental issues for invertebrate such as shell formation, settlement and metamorphosis. For instance, metamorphosis, which involves transformations of many organs during the transition from a free-swimming larva to a benthic juvenile that usually occurs within a short period of time. Metamorphosis is also the critical phase in terms of mortality in the life cycle for bivalves [2]. In the past years, progresses have been made in the mechanism of metamorphosis. Related genes and proteins have been identified and their functions were analyzed on abalones, ascidians, corals and so on. The spatial expression patterns of five anterior *Hox* genes during larval development of the abalone *Haliotis asinina* were reported and *Hox* genes were suggested to play an important role in gastropod shell formation [3]. The expression of Hemps, an EGF-like signaling peptide required for the induction of ascidian *Herdmania* metamorphosis, increases in competent larvae, and gene expression patterns in pre-competent and competent stages are markedly different [4]. Gene expression microarray analyses were performed in the scleractinian coral to elucidate the molecular mechanisms underlying coral metamorphosis and early stages of calcification [5]. However, studies were still limited and not deep enough to form integrated theories about the developmental biology of invertebrate. Because this field has long suffered from the challenges of lack of genomic resources such as genome or transcriptome sequences.

Oysters are widely distributed along the coast of China and are the most important commercial shellfish group cultured in China. The annual production of oysters was about 3.5 million tonnes in 2009 and accounted for more than 20% of mariculture total production in China [6]. Due to its economic value and ecological role, in recent years many studies have been carried out in its genetics [7], [8], breeding [9]–[11], disease control [12], [13] and so on. Fujian oyster, *Crassostrea angulata*, is the main oyster species in coastal river mouths and estuaries of southern China, ranging from Zhejiang Province to Hainan Province. The production for Fujian oyster usually accounts for about 50% of total oyster production in China. A recent study found that *C. angulata* should be considered as a subspecies of *C. gigas* by sequencing 16 S rRNA and COI genes [14].

The sequencing and analysis of expressed sequence tags (ESTs) has been a primary tool for the discovery of novel genes in terrestrial animals, especially in non-model species for which full genome sequences are not currently available. The recent high-throughput sequencing technologies, which can effectively speed up genomic studies on non-model animals, provide a great potential for bivalve research. The increased throughput of next-generation sequencing technologies, such as the massively parallel 454 pyrosequencing, enables the rapid generation of transcriptomes for non-model species and allows increased sequencing depth and coverage, while reducing the time, labor, and cost. According to this technology, transcriptome has been used in shellfish research in the past several years for solving some basis scientific problems, such as immune response, adaption under environmental pressure, and shell formation on *C. gigas* [15], *Mytilus galloprovincialis* [16] *Sinonovacula constricta* [17], *Laternula elliptica* [18], and *H. midae* [19].

By sequencing cDNA libraries for *Aplysia*, a well-established model organism for cellular and systems neural science, over 175,000 ESTs have been identified, and 19,814 are unique neuronal gene products which represent 50%–70% of the total *Aplysia* neuronal transcriptome [20]. High throughput EST pyrosequencing was also undertaken on the calcifying mantle, combined with a proteomic analysis of the shell for *Pinctada margaritifera*, to increase genomic resources and identify shell matrix proteins implicated in biomineralization [21]. *De novo* transcriptome sequencing was performed on *Patinopecten yessoensis* using the 454 GS FLX platform [22]. Approximately 25,000 different transcripts and a large number of SSRs and SNPs were identified. Transcriptome characterization of the South African abalone *H. midae* using sequencing-by-synthesis was also conducted and many gene families involved in immune response were identified [19].

Oyster is an emerging model in evolution and development, ecology and conservation of lophotrochozoan. In the present research, we performed *de novo* transcriptome sequencing for *C. angulata* at eight different early developmental stages using the 454 sequencing technology. Deep-coverage EST database was provided and a considerable amount of sequence data was annotated, while some functional genes involved in growth and early development were identified and quantified. These information would represent a valuable resource for genetic research and genomic studies on oysters.

## Results and Discussion

### Sequencing and Assembly

By virtue of sequencing from the 454 GS-Flx platform, the single run produced 566,917 reads with an average length of 319 nucleotides, which come from eight different developmental stages of the oyster. After adaptor trimming process, 555,215 reads still remained, totaling 173 Mb, with an average length of 309 nucleotides, which indicated that 97.9% of reads from pyrosequencing might be valid for further analysis. Since too short sequences (<60 bp) might represent contaminants or artifacts during sample preparation, they were removed, resulting in 540,423 (95.3 %) remained reads with an average length of 317 bp and 172 Mb in total (Table 1).

**Table 1:** Summary statistics of the ESTs generated from the Fujian oyster through pyrosequencing.

Features	Values
Number of raw sequences	566,917
Total bases of raw sequences	180,770,245 bp
Average length of raw sequences	319 bp
N50 of raw sequences	367 bp
Number of trimmed, size selected sequences	540,423
Number of scaffolds	1,732
Number of contigs	10,462
Number of singletons	108,592
Number of unigenes	120,786

**Figure 1:**
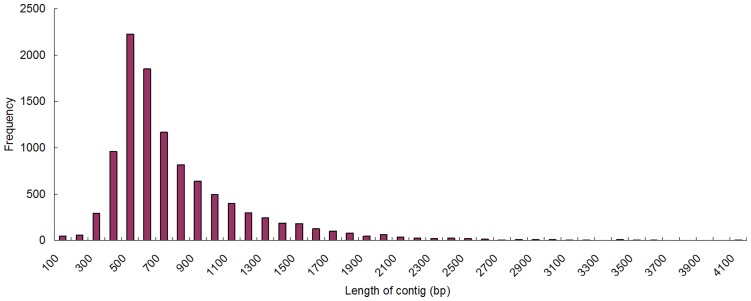
Distribution of the lengths of the ESTs for Unigenes of Fujian Oyster by 454 sequencing.

Contig construction of the trimmed, size selected reads using Newbler assembly software and scaffold construction process brought about 10,462 contigs and 1,732 scaffolds, with 108,592 sequences remained as singletons. The average length for contigs, scaffolds and singletons were 723 bp, 795 bp and 275 bp, respectively. Of contigs, 6,878 were more than 500 bp, and 1,907 more than 1 kb. The size distribution of these contigs is shown in Fig. 1. These results demonstrated that rapidly capturing a large portion of the *C. angulata* transcriptome by 454 pyrosequencing is effective. Singletons were preserved because some reads are likely to be fragments of original transcripts with low level expression, which may have corresponding similarities against known genes. This hypothesis can be confirmed by either PCR validation or analysis of sequence similarity. After these processes, the number of unigenes was determined, with all contigs, scaffolds and singletons. These assembled sequences, with the number of 80,852, whose lengths were more than 200 bp, were submitted to NCBI Transcriptome Shortgun Assembly sequences database (Accession no: JT981479–JT999999, JU000001–JU062331). However, scaffolds which had unknown gaps and those whose lengths were less than 200 bp, were deposited in Figure 1.

These processes including adaptors trim, size selection, assembly and contig joining, ensured the precision of subsequent data analysis, and also were considered to reduce redundancy among the sequences. These steps facilitated the analysis of gene name annotation, GO annotation, etc. Annotation of sequences helped us identify a particular gene of our interest rapidly, and provided more convenience for the studies of sequence polymorphism.

### Gene Annotation

Using Blastx program, sequences were first blasted against the smaller but well-annotated Swiss-Prot protein database, and then those reads having no significant hits were blasted against the nr database. Gene names and descriptions were assigned to those assembled sequences which had best blast matches (E<0.001) to subjects in the database. This process successfully annotated 25,654 sequences (21.2%) with known genes in the whole dataset, of which 18,526 were more than 300 bp in length and 1,469 were more than 1 Kb. Compared to other species, such as 16.8% of *H. midae* [19], 17% of *L. elliptica* [26], 24% of *R. philippinarum* [27], 28% of *P. yessoensis* [22], the annotation of *C. angulata* gained more descriptive information. Of all annotated sequences, 21,021 unique gene names were found, providing an estimated number of different genes expressed in the libraries.

**Figure 2:**
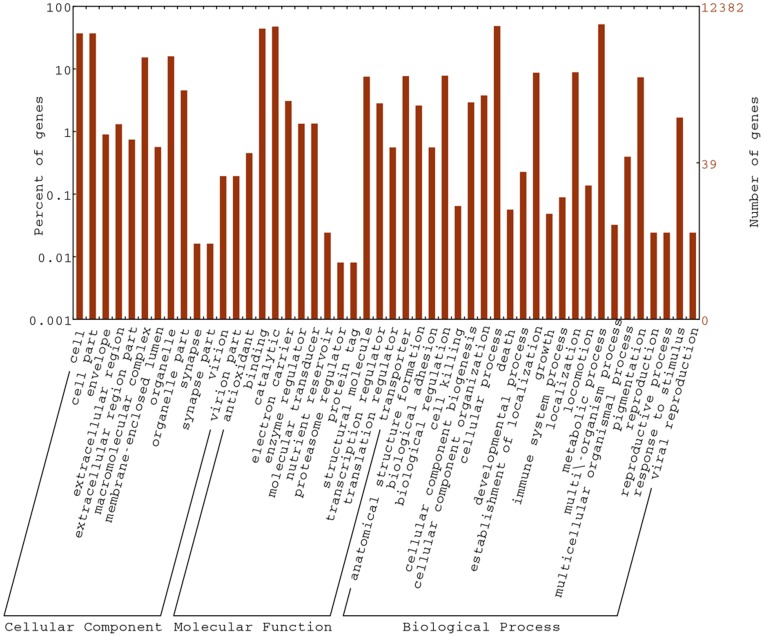
Gene Ontology (GO) analysis of transcriptome associated with oyster of early development. (The percentage and distribution of top-level GO-terms were portrayed in the three categories: Biological process, Cellular component and Molecular function.).

GO terms were assigned to 21,498 assembled sequences in view of sequence similarity with known proteins in UniProt-TrEMBL database with rich GO terms. Above all, the gene name annotation and GO annotation provided us an overview of each assigned sequence and number of genes related to a specific process. For example, GO annotation showed that 142 sequences were associated with developmental growth, 611 sequences were related to cell proliferation and 322 sequences belonged to immune response. The detailed distribution of genes in main ontology is illustrated in Fig. 2. From the statistics, we found that the composition and the distribution of assigned GO terms of molluscan species shared high similarities [23], [28]–[30], indicating the similar genes or metabolic pathways. Therefore, GO annotation provides us another new method for analyzing unknown sequences, improves the investigation of specific procedure, cellular structures and protein functions on those processes.

To gain overall information on oyster larval transcriptome, all the unigenes were analyzed in depth. Through blasting against unigenes with an E-value lower than 1e-9, 20 most commonly expressed sequences were extracted and listed in Table 2. From the level of gene expressions, arginyl-tRNA gene and peptide elongation factor gene were highly expressed, suggesting that protein synthesis process was active during larval stages. In addition, myosin gene was also much expressed, which may be related to the development of muscle on the oyster. Other housekeeping genes such as NADH dehydrogenase genes and cytochrome c oxidase genes all had relationship with biological process and protein transfer, which were also found in Antarctic krill *Euphausia* [26] and seahare *Aplysia californica* [29]. In addition, it is worth mentioning that transcription factor AP-2 gene was also important on reproduction and development at the larval stages. This protein acts as a sequence specific DNA-binding transcription factor recognizing and binding to the specific DNA sequence and recruiting transcription machinery, playing a vital role in protein activities. Comparing with transcription factor AP-2, importin is a type of protein that moves other protein molecules into the nucleus by binding to a specific recognition sequence, called the nuclear localization signal (NLS). Importin has two subunits, importin α and importin β. Of these, importin α binds to the NLS of the protein to be imported to the nucleus, whereas importin β helps in the docking of the importin heterodimer-bound protein to the nuclear pore complex, which ensures the correctness of protein transportation and efficiency of protein functioning.

**Table 2:** Most commonly expressed sequences with associated Blast matches of *C. angulata.*

Seq ID	No.of reads	Function	Species sources	E Value
SeqIndex73248	6,826	Arginyl-tRNA synthetase-like	*Saccoglossus kowalevskii*	6.00E-18
SeqIndex76912	2,213	elongation factor 1 alpha	*Crassostrea gigas*	0
SeqIndex2226	1,936	No significant blast hits		
SeqIndex68939	1,828	NADH dehydrogenase subunit5	*Crassostrea angulata*	0
SeqIndex31482	1,806	No significant blast hits		
SeqIndex31515	1,625	No significant blast hits		
SeqIndex74248	1,524	importin 7-like	*Saccoglossus kowalevskii*	3.00E-24
SeqIndex3932	1,423	28 S rRNA	*Crassostrea gigas*	1.00E-16
SeqIndex40717	1,364	No significant blast hits		
SeqIndex59590	1,246	No significant blast hits		
SeqIndex31626	1,204	No significant blast hits		
SeqIndex64013	1,178	Transcription factor AP-2 alpha	*Mus Musculus*	7.00E-09
SeqIndex58940	1,161	NADH dehydrogenase subunit2	*Crassostrea angulata*	2.00E-11
SeqIndex31572	1,144	No significant blast hits		
SeqIndex65672	1,121	NADH dehydrogenase subunit1	*Crassostrea iredalei*	4.00E-109
SeqIndex61327	1,115	Cytochrome c oxidase polypeptide 3	*Crassostrea angulata*	1.00E-132
SeqIndex59336	1,094	Isolate Cangtaiwh-9 mitochondrion	*Crassostrea angulata*	0
SeqIndex58602	1,094	No significant blast hits		
SeqIndex58021	1,083	No significant blast hits		
SeqIndex61965	1,027	Myosin essential light chain	*Crassostrea gigas*	4.00E-79

### Functional Genes Related to Growth and Early Development

Understanding the mechanisms on shell formation, settlement and metamorphosis of shellfish is currently an active research area. In the present study, from the early development of the Fujian oyster transcriptome expression profiles, contigs with similarity to the vitellogenin amino acid sequence were selected (Table 3). Six unique sequences related to growth and early development were detected, such as adrenergic receptors, dopamine receptors, epidermal growth factor receptor (EGFR), insulin-like growth factor 1 receptor (IGFR), insulin-induced protein, follistatin precursor. Forward and reverse primers designed based on the DNA-sequence of these contigs is shown in Table 4, while the length of the amplified fragment and the annealing temperature used during PCR is included.

**Table 3:** Contigs from *C. angulata* with similarity to the vitellogenin amino acid sequence.

Contig ID	Length (nt)	ORF	Accession	E value	Top match
C_13394	786	+2	NP_001116897	2e-21	adrenergic beta-2-receptor [*Xenopus (Silurana) tropicalis*]
8_50405	391	+2	CAA06536	5e-27	dopamine D1/beta receptor [*Branchiostoma lanceolatum*]
C_24709	791	+3	ADK98534	8e-41	epidermal growth factor receptor [*Bos taurus*]
7_37000	1,363	−3	3I81_A	2e-97	Insulin-like growth factor 1 receptor [*Homo sapiens*]
5_51106	311	+2	XP_002730421	4e-10	Insulin-induced protein [*Saccoglossus kowalevskii*]
C_23983	650	+1	XP_783040	4e-10	Follistatin precursor [*Strongylocentrotus purpuratus*]

The adrenergic receptor was suggested to mediate metamorphosis of the oyster *C. gigas* by the pharmacological and ecological experiments [31]. Coon and Bonar [32] also have brought forward a model including two pathways in controlling the settlement and metamorphosis of the oyster larvae. The model for settlement and metamorphosis indicated that control of settlement behavior appears to be via a dopaminergic receptor-mediated neural pathway, while control of morphogenesis is through an adrenergic receptor-mediated pathway [32]. But until now research about settlement and metamorphosis was limited in the field of pharmacology and ecology.

**Figure 3:**
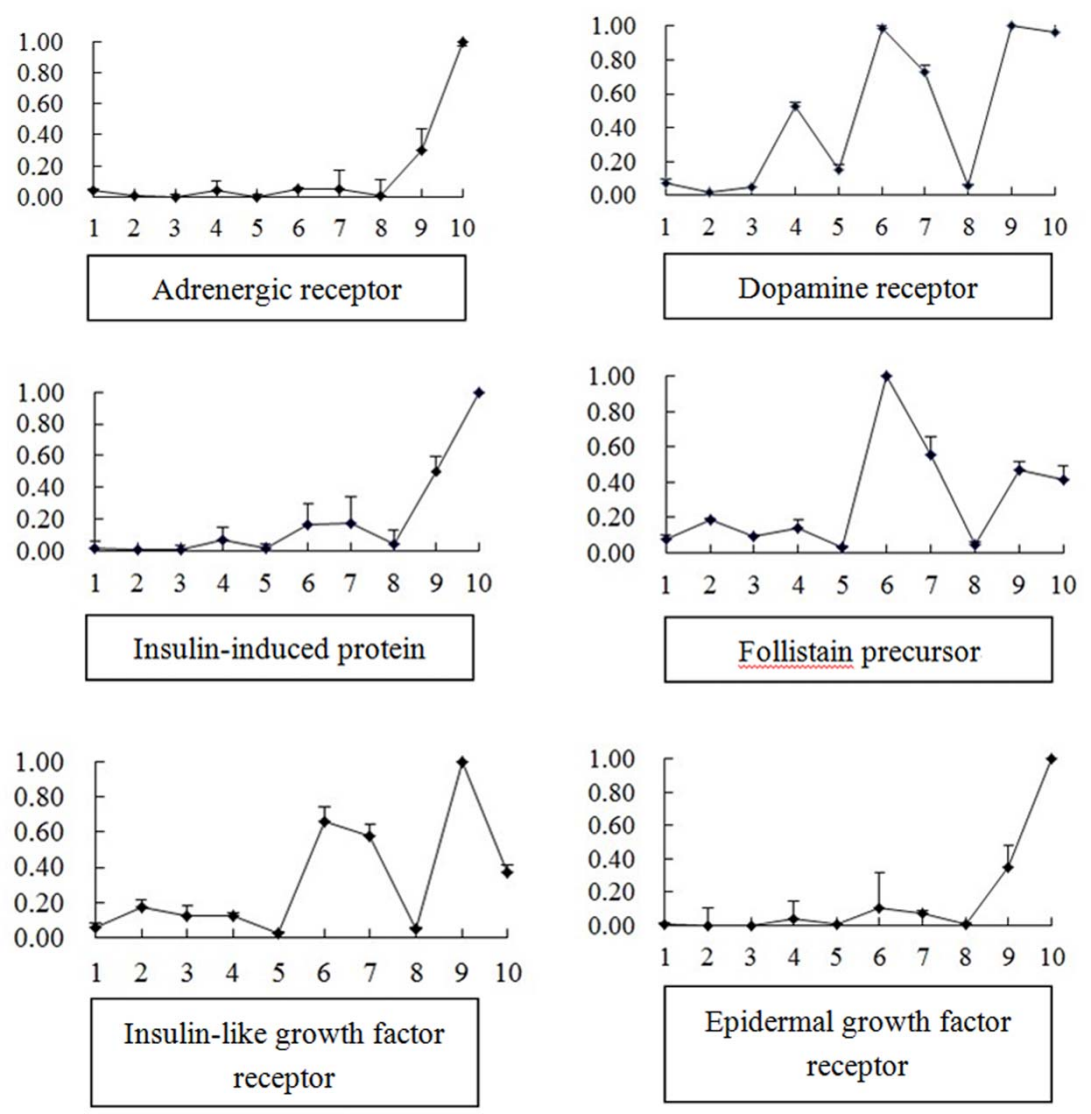
Gene expression in different developmental stages of Fujian Oyster (1: Eggs; 2: Gastrula; 3: Trochophore; 4: D-larvae; 5: Umbo larvae; 6: eye spot larvae; 7∶0.5 h after settlement; 8∶6 h after attachment; 9∶9 h after attachment; 10∶24 h after attachment).

As shown in Fig. 3, before settlement, expression of dopamine receptor and adrenergic receptor rise steadily as oyster developed. The expression reached the highest level in eye spot larva, while the expression decrease was detected in umbo larva. After settlement, expression of dopamine receptor and adrenergic receptor both declined to the lowest level at 6 h after settlement, and then the expression increased again at 9 h and 24 h after settlement. It suggests that oyster larvae have a promoting sensitivity to the exterior environment during early development and then ready for receiving metamorphosis stimulation at eyespot larvae stage. The exogenous signal, which triggers a distinctive pattern of settlement, is converted into dopamine with the larval body and acts through dopaminergic receptors. The higher expression levels in the eyespot larvae indicated that the competent larvae can begin the process of metamorphosis after meeting the appropriate conditions. The expression pattern found in the present study confirmed the results of the Coon and Bonar model [32].

Some important genes involved in growth were revealed to be differentially expressed among the 10 different developmental stages. For the four growth-related genes, EGFR, IGFR, insulin-induced protein, follistatin precursor, the expression pattern was nearly the same. The expression was kept at the low level before settlement and then increased in eye spot larvae and 0.5 h after settlement. The value decreased to the lowest level at 6 h after settlement and then increased quickly. Insulin-like biological effects involve a variety of molluscan cell types, including those from the nervous ganglia, mantle, digestive gland, gonad and haemolymph [33]–[34]. Insulin-related peptides were also shown to stimulate protein synthesis in haemocytes of *H. tuberculata* [35]–[36]. Follistatin also known as activin-binding protein is the binding and bioneutralization of members of the TGF-β superfamily, with a particular focus on activin, a paracrine hormone. This gene is proved to involve in muscle growth and development in catfish [37]. For oysters, there are more morphological changes and organ development before settlement. But after settlement, the juveniles are growing very quickly, while it is reasonable to explain the up-regulation for the follistatin gene.

Next generation sequencing is used for the large scale transcriptome sequencing on *C. angulata*. A considerable amount of sequence data was further annotated. The results obtained here provide a foundation for future genetic studies exploring ways to optimize the commercial production of *C. angulata.*

## Materials and Methods

### Larvae Culture and Sample Collection

The oyster brood stocks were collected from coastal waters of Zhangpu in Fujian Province. Parent oyster culture, spawning and larvae rearing were conducted as described by Evans and Langdon (2006). Planktonic larvae were cultured in the 4 m×6 m×1.8 m tanks at 25–28°C with the density of 200/mL. Mixed microalgae of *Dicrateria zhanjiangenis, D. zhanjiangenis* and *Platymonas subcordiformis* were used as diet for the larvae. The oyster for the eight major developmental stages, viz., 1^st^ and 2^nd^ polar body, gastrula, trochophore, D-veliger, pediveliger, perna viridis, postlarvae (settled for 30 min) and spat (settled for 2–8 days) were collected. The collected samples were added with Trizol (Invitrogen) and frozen in liquid nitrogen quickly, and then stored at −80°C till use.

### RNA Purification, Reverse Transcription, and 454 Sequencing

The samples from each stage were used for RNA purification and sequencing. Total RNA was isolated by Trizol and RNA integrity was monitored by electrophoresis in 8% denaturing polyacrylamide gels. mRNA was purified through MicroPoly (A) Purist kit (Ambion).

cDNA samples were prepared following the protocol described in [23]. mRNA (500 ng) were used as templates to synthesize the first-strand cDNA using a SMART^TM^ PCR cDNA Synthesis kit (Clontech, CA, USA). Reaction volumes contained 5 × First-strand synthesis buffer 2 µL, 10 mmol/L dNTPs 0.5 µL, 0.1 mmol/L DTT 1 µL, 10 µmol/L Template-switch primer 1 µL, CDS/3′-*Bsg* I primer 1 µL, mRNA 3.5 µL, Reverse transcriptase 1 µL. The profile was as follows: 42°C for 90 min, 70°C for 10 min. 3′ SMARTIM CDS Primer II A primer was changed as CDS/*Bsg* I (5′- ATTCTAGAGGCCGAGGCGGC*GTGCAG*TTTTTTTTTTTTTTTTTT TVN -3′) in order to cut the Poly(A) tail. Double strands cDNA was then amplified using 5′cap-primer and CDS/Bsg I. Reaction volumes contained First-strand cDNA 1 µL, 10×Ex Taq PCR buffer 2.5 µL, 2.5 mmol/L dNTPs 2 µL, 10 µmol/L 5′cap-primer 1 µL, 10 µmmol/L CDS/3′-BsgI primer 1 µL, ExTaq (TaKaRa) 0.25 µL, ddH_2_O 17.25 µL. The PCR profile was 95°C for 1 minutes, (95°C 15 s, 65°C 30 s, and 68°C 6 mins) ×18 Cycles, and 72°C 5 mins. PCR products were purified by Axygen PCR Purification kit (Axygen). 0.1×NaAC and 2.5×absolute alcohol was added in the PCR products for concentration and precipitation. Finally, approximately 5 µg of cDNA was used to construct a 454 library. Roche GS-FLX 454 pyrosequencing was conducted by Meiji Biotechnology Company (Shanghai, China).

### Data Analysis of 454 Sequencing

The raw reads were first filtered since adaptors and short reads still existed in the library. All the trimmed reads whose lengths were over 60 bp were preserved for the further analysis. Valid reads were entered into Newbler assembler program to be assembled into contigs, and parameter settings were the recommended stringency of 40 bp overlaps with 90% similarity. After assembly, contigs and singletons were preserved to build large scaffolds, referred to Meyer’s perl script [23], based on the fact that reads without overlaps might belong to one unique subject, which should be concatenated with 10 ‘X’s between them in order to remove relative redundancy and determine the final unigene set. Similarity searches were done using BLASTx program with Swissprot and nr database. GO analysis was conducted by Blast2GO [24] and Wego [25]. In order to detect the detailed functional genes of the oyster, top expressed genes were investigated, which may reflect gene expression level of the oyster larvae to some extent. BLAST each EST (longer than 60) against Unigenes with blastn program, and calculate the number of hits of each subject. Top 20 genes expressed were then analyzed.

**Table 4:** Forward and reverse primers designed based on the DNA-sequence of contigs.

Contig ID	Primer sequence		Amplified fragment size	Annealing temperature (°C)
	Forward	Reverse		
C_13394	AGGATTCGGGTCAGGAG	TTGCGAGTTTGCGTTTAT	175	60
8_50405	GTCCACGCCAAGTCAATACCAAC	TGCAGCGAATTATGATCTAAAGG	109	61
C_24709	ATTTCAAGAGGGCGAGTT	CACAGGCAGGATCACATAC	156	63
7_37000	CATCGCTGTCACAGTATCGG	TTGTCACGCTCCACTTCC	171	62
5_51106	TGAGATGTATTGCCGTATT	CAGCCACCACAGTCCTAT	116	64
C_23983	AGATACCTGGATTCACCCC	TCGTCACTTCCGCAAACT	174	65

### Real-time PCR

Six contigs with similarity to the vitellogenin amino acid sequence were selected. Primers (listed in Table 4) based on target genes were designed using Primer Premier 5.0 software (PREMIER Biosoft International, CA, USA). PCR reaction volumes contained cDNA 1 ng, 10×PCR buffer 2.5 µL, dNTPs (2.5 mmol/L) 2 µL, Forward Primer (5 µmol/L) 1 µL, Reverse Primer (5 µmol/L) 1 µL, templete 1 µL, *Ex Taq* (TaKaRa) 0.2 µL, ddH2O 17.3 µL. The profile was as follows: 95°C 3 min, (95°C 15 s, 55°C 30 s, 72°C 20 s) × 40 Cycle, 72°C 5 min, with a final extension step of 10 min at 72°C. PCR products were then detected by 1.5% agarose gel.
